# Do cognitive performance and physical function differ between individuals with motoric cognitive risk syndrome and those with mild cognitive impairment?

**DOI:** 10.1186/s12877-020-01992-z

**Published:** 2021-01-09

**Authors:** Fang-Yu Cheng, Yuanmay Chang, Shih-Jung Cheng, Jin-Siang Shaw, Chuo-Yu Lee, Pei-Hao Chen

**Affiliations:** 1 Institute of Long-Term Care, Mackay Medical College, New Taipei City, Taiwan; 2 Department of Neurology, Mackay Memorial Hospital, No. 92, Sec. 2, Zhongshan N. Rd, Taipei, 10449 Taiwan; 3 Department of Medicine, Mackay Medical College, New Taipei City, Taiwan; 4 Department of Physical Therapy and Assistive Technology, National Yang-Ming University, Taipei, Taiwan; 5 Graduate Institute of Mechanical and Electrical Engineering, National Taipei University of Technology, Taipei, Taiwan; 6 Graduate Institute of Chemistry, Tamkang University, New Taipei City, Taiwan

**Keywords:** Motoric cognitive risk syndrome, Mild cognitive impairments, Cognitive performance, Physical function

## Abstract

**Background:**

Motoric cognitive risk syndrome (MCR) is defined by slow gait speed combined with subjective cognitive complaint. MCR is a predementia syndrome, similar to mild cognitive impairment (MCI). However, there is currently no study comparing the differences in cognitive performance and physical function between these two types of cognitive impairment. Thus, the aim of this study is to compare cognitive performance and physical function in individuals with MCR versus MCI.

**Methods:**

A total of 77 participants, free of dementia, were recruited from the neurological outpatient clinic of a medical center in Taiwan. Participants were separated into 2 groups, MCR (*n* = 33) and MCI (*n* = 44) groups, based on definition criteria from previous studies. The priority was to assign a diagnosis of MCR first, followed by MCI. Hence, “pure” MCI had no overlap with MCR syndrome. Cognitive performance, including executive function, attention, working memory, episode memory, visuospatial function, and language, were measured. Physical functions such as activities in daily living, the Tinetti Assessment Scale, and the Timed Up and Go test were also measured.

**Results:**

Executive function, attention, working memory, episodic memory and language were all significantly lower in the MCR group than the MCI group. Abilities related to physical function, including those measured by the Tinetti Assessment Scale and the Timed Up and Go test, were significantly lower in the MCR group than the MCI group.

**Conclusions:**

We noted that cognitive performance and physical function were lower in MCR individuals than MCI but without MCR syndrome. However, the conclusions were based on the enrollment procedure of participants prioritizes the MCR syndrome. Because of the overlap of MCR and MCI, future studies should use different enrollment strategies to further clarify the status of these two populations.

##  Background

The prevalence of dementia and its associated medical and long-term care costs are growing rapidly internationally as the global population ages [1]. Several studies have shown that cognitive decline, especially in the memory domain, is a common condition of aging and is also considered to be related to the occurrence of dementia [2]. Functional impairment in the cognitive and physical domains occurs with aging and makes elderly individuals vulnerable to adverse events such as disability, falls, or even death [3–5]. Therefore, it is important to identify and validate biomarkers for the early diagnosis and identification of populations that are at risk of dementia [6].

Mild cognitive impairment (MCI) is a condition that has been studied for 2 decades and corresponds to a state of cognitive function between that of normal aging and dementia [7]. The clinical diagnosis of MCI requires a precise evaluation process, including face-to-face consultations, a series of neuropsychological tests and functional performance tests. This entire assessment takes a substantial amount of time, requires trained medical professionals and is not easy to implement in the community. In the clinical MCI population, the annual conversion rate to dementia has been shown to range from 10–15% [8–10]. However, the conversion rate in community-dwelling populations is often substantially lower, ranging from 3.8–6.3% per year [11–13]. In addition, community-dwelling elderly individuals with MCI are an unstable group, as almost all of them exhibit a change in the functional category each year [13]. Some MCI individuals’ conditions remain stable or even return to normal [14]. This outcome might be incorrectly recorded because of limited resources and the time-consuming nature of the cognitive assessment [15]. Therefore, it may be necessary to expand or modify clinical risk assessments of dementia in community populations.

Several studies have shown that the simultaneous presence of cognitive impairment and gait disturbances is common in elderly individuals [16–18]. Additionally, these functional limitations may suggest early signs of dementia [19, 20]. Motoric cognitive risk syndrome (MCR) is new and was proposed by J Verghese, C Wang, RB Lipton and R Holtzer [21]; it is characterized by a slow gait speed [1 standard deviation (SD) below the mean age- and sex-specific gait speed] and subjective cognitive complaints. MCR does not require a lengthy comprehensive neuropsychological evaluation, making the assessment highly advantageous in detecting older adults in the community who are at high risk of dementia [21, 22]. In addition, recent studies have shown that MCR can be used to predict the occurrence of disability [23], falls [24] and death [3] in older populations. However, MCR was only recently proposed, and the application and characteristics of MCR are not yet well established.

As mentioned above, MCI and MCR are both predominant cognitive impairment syndromes. However, there are currently no studies comparing cognitive performance and physical function between individuals with these 2 types of cognitive impairment. Thus, the aim of this study was to compare cognitive performance and physical function in individuals with MCR versus those with MCI. MCR has been shown to have improved predictive validity for dementia [18] and falls [24] compared to its individual cognitive and motor components. Therefore, we assumed that older adults suffering from MCR have poorer cognitive and physical performance than do those with MCI. Identifying the differences in functional capacity between individuals with MCR and MCI may help us understand the difference between these two syndromes and predict dementia in elderly individuals.

##  Methods

###  Participants and study design

**Fig. 1:**
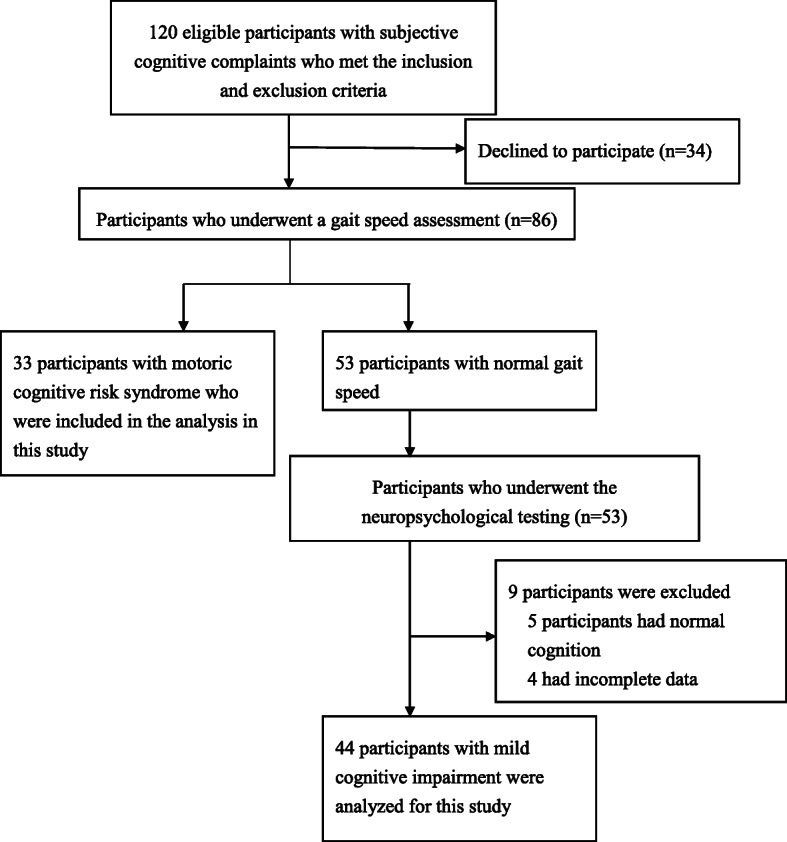
Flow chart showing the process of selecting subjects in this study

This study was a cross-sectional study conducted in Taiwan between April 2018 and August 2018. Participants were recruited from a medical center in Taipei. All participants met the following criteria: (a) aged 60 years or older, (b) could walk 10 meters independently, (c) were community dwelling, (d) had subjective cognitive complaints and (e) were not diagnosed with dementia. The presence of subjective cognitive complaints was determined by a ‘yes’ response to the memory item on the Geriatric Depression Scale [25, 26]. Dementia was diagnosed after all clinical and neuropsychological information presented at diagnostic case conferences were reviewed. The exclusion criteria were as follows: (a) unstable medical conditions, for example, the presence of major visual or hearing loss, (b) a recent or planned surgery leading to limitations in walking and interfering with participation in this study or (c) the consumption of any medications causing cognitive complaints during the past 3 months. A total of 86 participants provided informed consent, and the study procedures were approved by the Institutional Review Boards of Mackay Memorial Hospital (number: 18MMHIS005e). We confirm that all methods were performed in accordance with the relevant guidelines and regulations. The subject inclusion process for this study is depicted in Fig. 1 (*n* = 77). The participants’ age, sex, history of metabolic disease, and subjective cognitive complaints were obtained from patient interviews and medical charts.

###  Study assessment

The selected participants underwent comprehensive neuropsychological testing and physical activity testing with the Barthel Index, Instrumental Activities of Daily Living (IADL) scale, Tinetti Assessment Tool and Timed Up and Go (TUG) test.

A face-to-face neuropsychological assessment was performed using the Chinese versions of parts A and B of the Trail Making Test (TMT) [27], the category fluency test [28], digit recall forwards and backwards [29], the Chinese version of the California Verbal Learning Test [30], the short form of the Judgment of Line Orientation test [31], the Boston Naming Test [32], and the Chinese version of the Geriatric Depression Scale-15 (GDS-15) [33]. The TMT is a neuropsychological test of visual attention and executive function [34]. The category fluency test was designed to measure executive functions and language [35]. Digit recall forwards and backwards are the most widely used short-term verbal memory and executive function tests [29]. The Chinese version of the California Verbal Learning Test is designed to measure episodic memory for the Chinese-speaking population aged ≥ 50 years old [30]. The short form of the Judgment of Line Orientation test is a 10-item standardized test of visuospatial performance [36]. The Boston Naming Test is one of the most commonly used measures of confrontation naming in individuals with language impairments caused by diseases such as Alzheimer’s disease and dementia [32]. The Chinese version of the Geriatric Depression Scale-15 is a valid assessment tool used to evaluate the prevalence of depressive symptoms among the Chinese rural elderly people [33].

Functional status was assessed using the Barthel Index for self-care activities such as washing, dressing, grooming, using the restroom, and eating and the IADL scale for activities such as cooking, shopping, doing laundry, and maintaining household finances. Physical function was also assessed by the Tinetti Assessment Scale, which is a test that is easy to perform and measures balance ability and gait [37]. The gait speed and TUG tests were conducted with G-WALK® (BTS Bioengineering Corp., MA, United States), which is a wearable system used for the functional analysis of movement. This system comprises a portable inertial sensor that is attached to the S1-S2 vertebrae and records specific movements. When a subject walks, the sensor collects and transmits data to a laptop for analysis. The sampling rate of the system is 100 Hz. The validity and reliability of this system to assess movement performance has been well established [38]. Gait speed was evaluated during walking at a habitual speed. The participants were asked to walk 10 meters at a comfortable speed 3 times, and the average speed of the 3 trials was used for data analysis. For the TUG test, the participants were instructed to stand up from a chair, walk 3 meters, turn around, walk back to the chair, and sit down a total of 3 times. The time needed to complete the task was recorded, and the average time of the 3 trials was used for data analysis.

After completing all assessments, the participants were separated into two groups, the MCR (*n* = 33) and MCI (*n* = 44) groups, based on the criteria reported in a previous study [12, 21]. MCR was diagnosed if participants met all of the following four criteria [21]: (a) subjective cognitive complaints, (b) a slow gait speed, (c) the absence of dementia, and (d) a consistent level of independence in activities of daily living. Subjective cognitive complaints was defined as reported in the inclusion criteria [25, 26]. Based on previous studies, a slow gait speed was defined as a walking speed of one SD or more below the mean age- and sex-specific gait speed [21, 39]. MCI was diagnosed using the following criteria [40]: (a) subjective cognitive complaints, (b) objective cognitive impairment in one or more cognitive domains, (c) preserved activities of daily living, and (d) the absence of a diagnosis of dementia. In this study, the priority was to assign a diagnosis of MCR first, followed by MCI. Hence, “pure” MCI had no overlap with MCR syndrome which indicated that all participants in the MCI group did not have slow gait speed [1 standard deviation (SD) below the mean age- and sex-specific gait speed].

###  Statistics

All statistical analyses were performed using SPSS version 22.0 (SPSS, Inc., Chicago, IL, United States). The participants’ characteristics were summarized using the means and SDs or numbers, as appropriate. Between-group comparisons were performed using independent t-tests (continuous variables) or chi-square tests (categorical variables). In this study, the correlations were first established, and the factors correlating significantly with gait speed were further analyzed using a linear regression model. Pearson’s correlation coefficient was used to examine correlations between cognitive performance and gait speed. We considered correlation coefficients larger than 0.3 as meaningful correlations [41, 42]. In addition, because the Pearson’s correlations were examined 11 times, the significance level was corrected with the Bonferroni method (*p* = 0.005) to reduce the possibility of statistical errors. A linear regression model (stepwise strategy) was used to determine the contributions of different cognitive domains to gait speed. A p value of less than 0.05 indicated statistical significance.

##  Results

### Baseline demographic data

Seventy-seven subjects (males: 43; females: 34) participated in the study. Table 1 presents the characteristics of the participants in the MCR (*n* = 33) and MCI (*n* = 44) groups. The mean ages of the participants in the MCR and MCI groups were 69.3 ± 9.8 and 70.0 ± 8.8 years, respectively. There were no differences in the basic characteristics (age, sex, body mass index, and educational level) or the prevalence of a medical condition (hypertension, diabetes, hyperlipidemia, and heart disease) between the subjects with MCR and those with MCI. The participants with MCR had a lower gait speed than did the MCI participants (*p* < 0.001).

**Table 1:** Comparison of participants’ characteristics between the two groups

	Motoric cognitive risk syndrome (*n* = 33)	Mild cognitive impairment^a^ (*n* = 44)	*P* value
Age (years)	69.3 ± 9.8	70.0 ± 8.8	0.733
Sex (male/female)	17/16	26/18	0.643
BMI	24.4 ± 3.4	23.9 ± 3.3	0.480
Educational level (years)	7.6 ± 3.7	7.9 ± 4.3	0.764
Gait speed (m/s)	0.7 ± 0.1	1.0 ± 0.2	< 0.001
Hypertension (n)	19	30	0.351
Diabetes (n)	12	14	0.808
Hyperlipidemia (n)	19	29	0.485
Heart disease (n)	11	16	0.814

*Abbreviations*: *BMI* body mass index

^a^All participants in the MCI group did not have slow gait speed [1 standard deviation (SD) below the mean age- and sex-specific gait speed]

### Comparisons between MCR and MCI

The cognitive performance and physical function of the participants with MCR and MCI are shown in Table 2. Compared to the subjects with MCI, those with MCR had poorer executive function (TMT-A, *p* = 0.034; TMT-B, *p* = 0.043; category fluency test, *p* = 0.003), a poorer memory ability (digit recall backwards, *p* = 0.016; California Verbal Learning Test-short form, *p* = 0.030), poorer language function (Boston Naming Test, *p* = 0.027), and more depressive symptoms (GDS-15, *p* = 0.028). In addition, the Barthel Index and IADL results were similar between the two groups, but the subjects with MCR had significantly lower Tinetti gait and balance scores (gait, *p* = 0.010; balance, *p* = 0.028) and a longer TUG completion time (*p* < 0.001) than did the MCI group.

**Table 2:** Comparison of the participants’ cognitive performance and functional status between the two groups

	Motoric cognitive risk syndrome (*n* = 33)		Mild cognitive impairment^a^ (*n* = 44)	*P* value
*Executive function*				
TMT-A (s)	30.0 ± 16.3		21.9 ± 16.2	0.034
TMT-B (s)	75.2 ± 39.1		59.0 ± 34.6	0.043
Category fluency test	10.8 ± 3.3		13.4 ± 3.9	0.003
*Attention and working memory*				
Digit recall forwards	7.2 ± 1.4		7.3 ± 1.6	0.764
Digit recall backwards	3.5 ± 1.1		4.3 ± 1.7	0.016
*Episode memory*				
California Verbal Learning Test-short form	17.6 ± 5.0		20.1 ± 5.2	0.030
*Visuospatial performance*				
Judgment of Line Orientation	13.2 ± 4.1		13.8 ± 3.2	0.522
*Language*				
Boston Naming Test	20.9 ± 5.3		23.6 ± 4.9	0.027
*Depression*				
Geriatric Depression Scale-15	3.9 ± 3.7		2.1 ± 2.6	0.028
*Physical function*				
Barthel Index	96.1 ± 10.8		99.9 ± 0.8	0.057
IADL	20.7 ± 5.3		22.3 ± 3.9	0.150
Tinetti gait	10.9 ± 2.0		11.9 ± 0.6	0.010
Tinetti balance	14.2 ± 3.4		15.6 ± 1.1	0.028
Timed Up and Go test (s)	22.7 ± 13.4		13.4 ± 3.1	< 0.001

*Abbreviations*: *TMT* Trail Making Test, *IADL *Instrumental Activities of Daily Living

^a^All participants in the MCI group did not have slow gait speed [1 standard deviation (SD) below the mean age- and sex-specific gait speed]

### Association of cognitive performance and gait speed

Table 3 shows the correlation results. The scores of the category fluency test, digit recall backwards test and short form of the California Verbal Learning Test were positively correlated with gait speed (*r* = 0.370–0.449, *p* < 0.005). The TMA-A scores were negatively correlated with gait speed (*r* = -0.376, *p* < 0.005). According to the regression models (Table 4), executive function was the most important factor in determining gait speed (Model 1: F = 14.02, *P* < 0.001, effect size f^2^ = 0.21, statistic power = 0.98; Model 2: F = 10.91, *P* < 0.001, effect size f^2^ = 0.33, statistic power = 0.99).

**Table 3:** Correlation coefficient between cognitive performance and gait speed

	Gait speed, r	*P* value
Age (years)	-0.219	0.056
Educational level (years)	0.171	0.138
*Executive function*		
TMT-A (s)	-0.376	0.001*
TMT-B (s)	-0.324	0.005
Category fluency test	0.449	< 0.001*
*Attention and working memory*		
Digit recall forwards	0.172	0.135
Digit recall backwards	0.370	0.001*
*Episode memory*		
California Verbal Learning Test-short form	0.391	< 0.001*
*Visuospatial performance*		
Judgment of Line Orientation	0.085	0.467
*Language*		
Boston Naming Test	0.305	0.007
*Depression*		
GDS-15	-0.292	0.010

*Abbreviations*: *TMT* Trail Making Test, *GDS* Geriatric Depression Scale. *p < 0.005

**Table 4:** Summary of linear regressions

Variable	β	*p*
*Model 1*		
Category fluency test	0.413	< 0.001
R square	0.171	
Adjusted R square	0.159	
*P* value	< 0.001	
*Model 2*		
Category fluency test	0.342	0.003
Trail Making Test part A (s)	-0.283	0.012
R square	0.246	
Adjusted R square	0.223	
*P* value	< 0.001	

Variables eliminated from regression model 1: California Verbal Learning Test, Digit recall backwards, Trail Making Test part A

Variables eliminated from regression model 2: California Verbal Learning Test, Digit recall backwards

##  Discussion

In the present study, it was demonstrated that compared to individuals with MCI but without MCR syndrome, subjects with MCR are more likely to exhibit executive function, attention, memory, and language impairments. Our results also showed that physical functions, including gait and balance performance, are significantly lower in people with MCR than in those with MCI but without MCR syndrome. To the best of our knowledge, this is the first study to compare cognitive performance and physical function in individuals with two types of cognitive impairment, MCR and MCI. We also found that executive function is the primary factor associated with gait speed compared with attention, memory, visuospatial, and language performance.

A major contribution of this study to the literature includes the results of cognitive performance and physical function in individuals with MCR versus those with MCI but without MCR syndrome. Recent reports have shown that the simultaneous presence of cognitive decline and a slow walking speed can predict dementia [19, 20]. MCR is a novel concept and is defined as a heterogeneous clinical manifestation characterized by the simultaneous presence of an objectively slow gait speed and subjective cognitive complaints [21, 43]. Individuals with MCI demonstrate cognitive impairment with minimal impairment in IADL [44]. Both conditions are linked to poor cognitive function and represent a stage of predementia [22]. However, currently, the information on the characteristics of MCR and the differences between MCR and MCI are still limited. In the present study, we found that compared to MCI but without MCR, MCR is linked not only to poorer physical function but also to poorer cognitive performance in tasks related to executive function, attention, working memory, episode memory, and language. This result indicates that compared with MCI but without MCR, MCR might lead to more severe overall functional deterioration in older adults. In a three-year longitudinal study, people with MCR were found to be at higher risk of developing dementia than were people with not MCR (hazard ratio: 3.27) [21]. A random effects meta-analysis reported that the relative risk of dementia was 3.3 in those with MCI versus age-matched controls 2–5 years later [45]. Although the conversion rates in these two groups are similar, the research designs and the participants included in these studies differed greatly. Since we found significant differences in cognitive performance and physical function between MCR and MCI patients, it is important that a large cohort study is conducted in the future to compare the rate of conversion to dementia between these two groups.

Based on the definitions of MCR and MCI, both are predementia cognitive disorders, and the factor that differentiates them is gait speed. Walking is a very common activity of daily living and is a consequence of multifactorial and multisystem (sensory, musculoskeletal, nervous, and cardiorespiratory) coordination. Executive function [16, 46] and attention [46, 47] are considered to be the most important cognitive domains affecting gait performance. Our research findings reported that people with MCR who had a slower gait speed also had poorer executive function, poorer attention and memory performance, poorer language function, and more depressive symptoms than did participants with MCI but without MCR syndrome. In addition, we also noted that executive function was the primary factor associated with gait speed compared with other domains of cognition, which is consistent with other research results [48, 49]. A growing body of evidence suggests that frontal subcortical circuits mainly control the speed of walking [50]. Moreover, compromised frontal lobe white matter was found to be associated with executive network functional impairments and slower walking speeds in elderly individuals [51, 52]. These brain areas are highly susceptible to white matter hyperintensities, microvascular damage, and neurodegenerative pathologies, which are common precursors of dementia [52]. In summary, brain regions and networks specifically control both higher-level cognitive function and gait performance and explain the relationship between a slow walking speed and dementia pathologies.

Regarding physical function, our results showed that the mean Tinetti Assessment Scale score in subjects with MCR was significantly lower than that in people with MCI but without MCR, indicating that individuals with MCR not only walk slowly but also have poor balance. Moreover, compared to the MCI group, the MCR group needed more time to complete the TUG test (mean, MCI group = 13.4 seconds; MCR group = 22.7 seconds, *p* < 0.001). The TUG test is a reliable measure of functional activity that captures transfers, straight walking, and turning movements [53]. It has been reported that this test can be used to identify elderly individuals who are prone to falls (completion time of ≥ 14 seconds), with a sensitivity of 0.87 and a specificity of 0.87 [54]. This result demonstrates that our MCR group had a high-risk for falling, which is consistent with other research results [24]. As mentioned above, maintaining a normal well-balanced gait is a complex process requiring the efficient cooperation of multiple systems, such as those controlling motor, cognitive, and sensory processes [16, 20, 55], and the inability to have a normal gait can lead to falls. We therefore suggest that MCR is an effective screening tool for the risk of falls in older populations.

According to our results, people with MCR have poorer cognitive function and physical function than do MCI but without MCR patients, indicating that MCR might lead to more severe overall functional deterioration than MCI in older adults. Previous study indicated that slowing gait occurs approximately a decade prior to MCI onset [56] MCR does not require a time-consuming comprehensive neuropsychological assessment, unlike MCI, which is convenient for the clinical evaluation of the risk of dementia in community-dwelling older populations. In addition, compared with people with MCI, people with MCR might have a higher risk of falling, based on the completion time for the TUG test. There are some study limitations that should be addressed in the present study. The inclusion of a relatively small number of participants and the enrollment procedure of participants prioritizes the MCR syndrome made it impossible to perform any subgroup analyses with sufficient power, such as an MCI subtype comparison. Studies with a larger sample size should be conducted in the future to confirm our results and to conduct subgroup analyses. Besides, previous study indicated that slowing gait occurs approximately a decade prior to MCI onset [56], which suggests that the diagnosis of MCR, based on slow gait speed and subjective cognitive complaints, might precede the diagnosis of MCI. However, our result supported the direct opposite, which might be related to the enrollment procedure with a priority for the MCR group and the recruitment place was in the medical center. The elderly with subjective cognitive complaints who came to the medical center were usually in more serious condition than the elderly in the community. Future research should expand the enrollment of participants to the elderly in the community. We also used a cross-sectional study design, which prevented us from investigating changes in cognitive and physical function in the two groups over time. To better understand the actual interaction between cognitive and physical functions in aging populations that are at high risk of dementia, a large cohort study is warranted and encouraged. Moreover, it should be noted that since MCR is defined as a condition characterized by a slow gait speed in the presence of subjective cognitive complaints in elderly people not diagnosed with dementia or mobility impairments, the MCR subjects included in this study may or may not have met the MCI diagnosis. In this study, we did not exclude MCR subjects with MCI, which may have affected our results. However, the definition of MCR does not include conditions that can exclude subjects with MCI [21]. Therefore, the characteristics and differences between MCI and MCR need further clarification.

## Conclusions

This study suggests that cognitive performance and physical function are lower in MCR individuals than in MCI but without MCR individuals and that executive function, compared with other cognitive domains, is the primary factor associated with gait speed. The significant differences between these two groups suggest that MCR leads to more severe overall functional impairment than does MCI in older adults. However, the conclusions were based on the enrollment procedure of participants prioritizes the MCR syndrome. Because of the overlap of MCR and MCI, future studies should use different enrollment strategies to further clarify the status of these two populations. Additional studies on the mechanisms of MCI and MCR and subsequently, longitudinal studies on these predementia syndromes would be worthwhile.

## Data Availability

The datasets used and/or analysed during the current study are available from the corresponding author on reasonable request.

## Abbreviations

MCR: Motoric cognitive risk syndrome
MCI: mild cognitive impairment
SD: standard deviation
IADL: Instrumental Activities of Daily Living
TUG: Timed Up and Go
TMT: Trail Making Test
GDS-15: Geriatric Depression Scale-15
